# “It Depends on Where You Are and What Job You Do”: Differences in Tobacco Use across Career Fields in the United States Air Force

**DOI:** 10.3390/ijerph19148598

**Published:** 2022-07-14

**Authors:** Tori L. Horn, Kathleen J. Porter, Kinsey N. Pebley, Rebecca A. Krukowski, Melissa A. Little

**Affiliations:** 1 Department of Psychology, University of Memphis, Memphis, TN 38152, USA; thorn@memphis.edu (T.L.H.); knpebley@memphis.edu (K.N.P.); 2 School of Medicine, University of Virginia, Charlottesville, VA 22903, USA; kjporter@virginia.edu (K.J.P.); wae4mq@virginia.edu (R.A.K.)

**Keywords:** tobacco, military, career

## Abstract

While tobacco use within the military is often discussed as being homogenously part of U.S. military culture, literature from civilian populations highlights that tobacco use varies by career field (e.g., “white collar” vs. “blue collar”). The objective of this qualitative study was to compare tobacco use by career fields in the U.S. Air Force. Airmen, Military Training Instructors, and Technical Training Instructors participated in 22 focus groups across five major Air Force Technical Training bases. Focus groups were conducted in-person using semi-structured interview guides and were audio-recorded. A conventional content-coding approach was used to code transcripts. Participants described substantial variation across the careers, which was attributed to social norms and the nature of jobs. Individuals in careers that spend most of their time outside were more likely to permit tobacco use. Conversely, tobacco use was seen as stigmatized in medical fields. Additionally, smokeless tobacco was identified as popular in certain careers because it could be used covertly on the job. Findings suggest that a one-size-fits-all approach to reducing tobacco use through policies and programs may not reflect the realities of military tobacco use. These findings may provide insights into other branches of the U.S. military with similar career fields.

## 1. Introduction

While tobacco use in the general population in the United States (U.S.) has declined [1], tobacco use remains high among U.S. military personnel [2,3,4]. Among newly enlisted Airmen (called such regardless of sex or gender identity) without prior service in the U.S. Air Force, approximately 15.3% reported currently using electronic cigarettes (e-cigarettes), 5.9% used cigarettes, 5.8% used cigars/cigarillos, 4.8% reported tobacco or snus use, and 2.6% reported hookah use [2]. The military is a diverse entity, with individuals holding various careers within the military and performing a wide range of job duties. Previous literature suggests differences in tobacco use among various career fields [1,5,6], but little is known about whether these differences hold true in the military. By understanding the unique behaviors and needs within different careers in the military, we will be able to tailor prevention and intervention efforts among different groups, as we have for civilian populations.

Previous literature with civilian populations has documented differences in tobacco use between occupations. For example, blue collar workers have been shown to be more likely to smoke than their white collar counterparts [1,6]. However, studies have been limited in their assessment of tobacco use within the context of specific occupations. One nationally representative study found that the use of any form of tobacco was most prevalent among blue collar workers, such as those working in construction (36.5%) and installation, maintenance, or repair occupations (37.2%) [5]. Tobacco use was lowest among white collar occupations such as education services (11.0%); arts, entertainment, and recreation occupations (17.4%); and finance and insurance occupations (17.6%). However, in the same study, estimates could not be obtained for the Armed Forces due to the small sample size [5]. Thus, research is needed to further explore tobacco use behaviors among the wide range of occupations available in the military.

Higher rates of smoking appear to be associated with the workplace setting, social culture, and the nature of the work [7]. For example, research suggests that differences in tobacco use are largely driven by the fact that many workers based outdoors or performing manual labor are less likely to be protected by smoke-free policies than indoor and desk workers [8,9]. Additionally, workers based outdoors and/or performing manual labor usually have frequently changing work environments, making it difficult to implement smoke-free policies in the workplace [10]. Additionally, the social culture of smoking, such as having more colleagues who smoke at work [6], viewing smoking as a social activity [11], and perceived coworker norms regarding smoking cessation [12] have all been associated with smoking prevalence in civilian populations. Further, in civilian populations, research has suggested that job-related stress may be another facilitator of tobacco use with a recent systematic review finding that job-related stress may lead to an increase in smoking behavior [13]. This may be particularly salient among military personnel given the heightened levels of work-related stress that they experience (e.g., deployment and combat [14]; work overload, relationships with superiors [15]).

### The Current Study

However, little is known about the how tobacco use differs by career fields in the military. Given that military personnel work in a variety of career fields, the high rates of tobacco use observed among this population may not be uniform across occupations. Thus, military personnel working in certain careers may need targeted tobacco control policies or resources to prevent initiation or promote cessation. Therefore, in order to develop effective tobacco control programs and policies, it is necessary to first gain a deeper understanding of modifiable risk and protective factors in workplaces that are associated with tobacco use. The present qualitative study sought to examine differences in perceptions of tobacco use and product preferences between career fields within the U.S. Air Force.

## 2. Materials and Methods

Data for the current study were collected as part of a larger qualitative study exploring factors predicting tobacco use among Airmen during Technical Training [16]. Study procedures were approved by the 59th Medical Wing Institutional Review Board. Technical Trainees, Military Training Leaders, and Technical Training Instructors from the five largest Technical Training schools (Fort Sam Houston, Goodfellow, Keesler, Lackland, Sheppard) were recruited into this study. The majority of non-prior service, enlisted Airmen undergo Technical Training (i.e., advanced job skills training) at one of these five bases. Military Training Leaders are active-duty supervisors of Technical Trainees, who ensure they are where they are supposed to be and dispense disciplinary action. Technical Training Instructors provide direct instruction to Technical Trainees related to their career field and can be in active duty or civilians.

### 2.1. Focus Group Procedures

Between July 2018 and February 2019, 22 focus groups with Airmen (*n* = 10 focus groups with 83 participants), Military Training Leaders (*n* = 7 focus groups with 48 participants), and Technical Training Instructors (*n* = 5 focus groups with 33 participants) were held. Technical Trainees were recruited during their last week of Technical Training. MTL and TTI volunteers were recruited by the senior MTL at each base during this same period; thus, Military Training Leaders and Technical Training Instructors were responsible for the Trainees who were also participants in the focus groups. Participants had to be at least 18 years of age; however, both individuals who used tobacco and did not use tobacco were eligible to participate. Among focus group participants, 78% identified as men and 72% reported tobacco use.

Focus groups were facilitated by pairs of trained non-military researchers, who fulfilled either the role of the moderator or the note-taker. To promote an open and unbiased environment, focus groups were held in a private room on base without supervisory personnel present. Participants were also provided with food. A waiver of consent was obtained for the study to allow the focus groups to remain anonymous, which is critical within military samples as superiors can request to see the data at any time and Airmen report engaging in behaviors inconsistent with military policy can receive disciplinary action including discharge from service. Therefore, demographic characteristics were not collected to preclude the identification of particular individuals. At the start of each focus group, the researchers introduced themselves and the study, answered any questions about the study, and asked permission to audio record the focus groups. Focus groups lasted approximately 45 min and had an average of seven participants (ranging from 4 to 11 participants).

The researchers followed semi-structured focus group protocols that captured perceptions and experiences related to individual tobacco use, facilitators and deterrents of tobacco use on base, and strategies to reduce tobacco use among Technical Trainees. Given the different experiences among participants, focus group protocols were tailored for Airmen, for Military Training Leaders and for Technical Training Instructors. The focus group protocols are described in detail elsewhere [16].

### 2.2. Data Analysis

Focus group audio tapes were transcribed by Datagain. Transcripts were cleaned by members of the research team. The research team used NVivo (v12) [17] software to manage the inductive content coding process [18]. Transcripts were initially coded as part of the primary qualitative analysis using an initial codebook consisting of facilitators and deterrents to tobacco use among Technical Trainees at personal, interpersonal, and environmental levels, which were identified through the literature and moderator notes [16]. During the coding process, trained research staff identified and discussed potential emergent codes. A frequently identified emergent code was “tobacco use varies by career field.” Due to its frequency, the research team felt this code, which was defined as a description of tobacco use and/or its facilitators or deterrents within the context of a specific military job, warranted additional exploration outside the primary analysis.

Following completion of primary coding, two researchers (KJP, RAK) reviewed passages coded as “tobacco use varies by career field” to ensure they reflected the code definition and to identify emergent sub-codes reflecting descriptions of perceived differences of tobacco use between career fields and descriptions of tobacco use within specific types of career fields. We identified specific categories of career fields by first identifying the career fields present at each of the included bases. Then, we reviewed the coded passages for identifiers that would suggest the career field of the speaker and/or the focus of the passage (e.g., security forces, cops, flight line, crew chief, shop, medic(al), intel). Depending on the number of times a specific career field was mentioned and the ability to specifically identify the career field (e.g., “shop” and “crew chief” could refer to multiple maintenance and flight line careers), specific career fields were collapsed into categories. This process identified the following four career field categories: outdoor/hands-on, indoor/desk, medical, and security forces. Definitions of these career fields are presented in Table 1.

As “tobacco use varies by career field” was identified during the coding process, relevant passages coded early in the process may have been missed. Therefore, to ensure the completeness of coding for this secondary analysis, a search of all transcripts for key terms reflecting the concept of career fields and the identified types of career fields was completed using the query feature within NVivo by one researcher (KJP). Passages containing these key terms were reviewed and, if they reflected the concept of tobacco use within career fields, were coded into “tobacco use by career field” and relevant sub-codes.

Two researchers (KJP, KNP) reviewed data coded into each of the identified career field-related codes to ensure fitness and identified emergent sub-codes reflecting perceptions of differences between career fields and perceived use, facilitators, and barriers to tobacco use within the career field. Passages were coded and reviewed.

## 3. Results

### 3.1. Broad Perceptions of Tobacco Use across Career Fields

As presented in Table 2, Airmen identified three main perceptions of differences between career fields. First, Airmen identified tobacco use as part of the Air Force and broader military culture. Second, they identified that tobacco use is not the same in all career fields and that “where you are and what job you do” impacts tobacco use. Third, they identified that tobacco use is perceived as higher among “outside” jobs, such as security forces positions, jobs on the flight line, or those related to aircraft maintenance, compared to “inside” or desk jobs, due to increased opportunities to use tobacco.

### 3.2. Descriptions of Tobacco Use by Career Field

Table 2 describes perceived use, facilitators, and deterrents to tobacco use within the four identified career fields: indoor/desk; medical; security forces; and outdoor/hands-on. Perceived tobacco use was consistently noted as high within security forces and outdoor/hands-on careers, while it was consistently noted as low among medical jobs. Tobacco use varied within indoor/desk careers, with use varying by specific role. Smokeless tobacco was frequently mentioned among those in the security forces and outdoor/hands-on careers.

**Figure 1:**
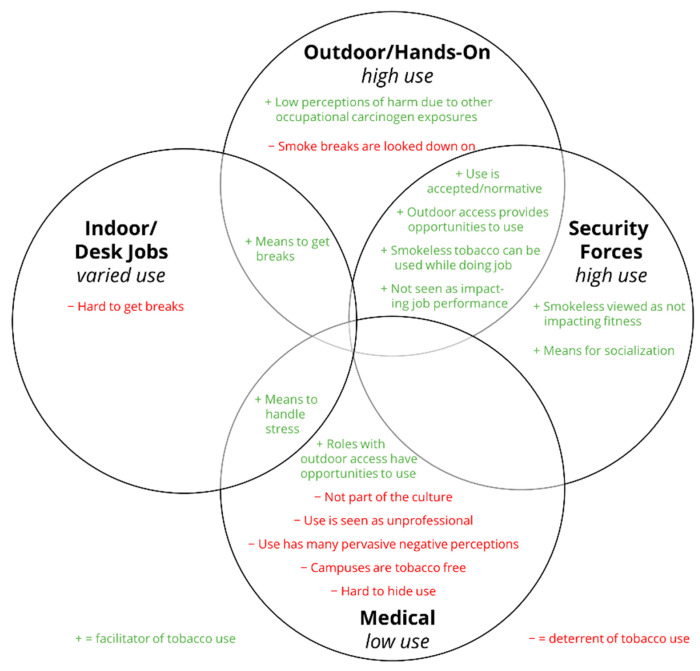
Facilitators and Deterrents of Tobacco Use by Career Field.

As shown in Figure 1 and described in Table 3, perceptions of facilitators varied by career field. Perceptions of tobacco use being normative or acceptable were noted as facilitators of tobacco use among those in security forces and outdoor/hands-on careers. Ease of access to opportunities to use tobacco were noted among career fields involving security forces, outdoor/hands-on careers, and medical roles that had access to outdoor spaces (e.g., technicians). These opportunities related to outdoor access and being able to use tobacco while performing their role functions. Stress was noted as a driver for tobacco use within indoor/desk careers and medical career fields. Opportunity for smoking breaks was noted as a facilitator among indoor/desk careers and outdoor/hands-on careers. Socialization was noted as a facilitator of tobacco use within security forces careers.

Similarly, deterrents for tobacco use varied among career fields. No specific deterrents were noted for security forces. Some deterrents were related to breaks, with breaks being hard to access for those with indoor/desk careers or those who ask for smoke breaks being viewed as lazy and looked down on in outdoor/hands-on careers. Medical careers had the most deterrents to tobacco use including use being viewed as not part of the culture; contrary to the intention of the field and, therefore, unprofessional; and high perceptions of negative impacts of tobacco use (e.g., health risks). Additional deterrents included medical campuses being tobacco-free, which reduced access, and the patient-facing nature of most medical jobs, which could make it hard to hide tobacco use (see Table 3).

## 4. Discussion

The present study aimed to examine differences in Airmen’s perceptions of tobacco use between career fields within the U.S. Air Force. Security forces and outdoor/hands-on career fields reported high perceived rates of tobacco use, while those in medical careers reported lower perceived rates of use. This is similar to quantitative reports of high tobacco use among civilians in blue collar careers (e.g., construction, manufacturing, and installation, maintenance, or repair occupations) and low tobacco use among those in white collar careers, such as health care and social assistance careers and those working in education services [5]. These results may speak to the descriptive norms of tobacco use within these respective career fields, or the perception of how many others engage in tobacco use behaviors. This is important given that descriptive norms were previously shown to influence engagement in tobacco use behaviors among military personnel [19].

Injunctive norms related to tobacco use, or the perception of how acceptable using tobacco would be among others and within different occupations may also play a role in use behaviors and the differences in use by career field. Airmen in outdoor/hands-on career fields described tobacco use as acceptable because others in these careers use tobacco while working. However, tobacco use in these careers was only viewed as acceptable if it did not interfere with work-related tasks (i.e., not appearing as slacking off at work). In contrast, Airmen in medical careers held negative views of tobacco use and perceived tobacco use as unprofessional and contrary to their work by others. Thus, perceptions about how others viewed tobacco use seemed to vary greatly by career field.

Differences in perceived tobacco use prevalence may also be attributable to the practicality of use, as evidenced by the differences in accessibility described by Airmen. Among security forces and outdoor/hands-on careers, smokeless tobacco was described as the most commonly used tobacco product, perhaps because Airmen in these career fields can use it discretely while on the job. Use of tobacco products is restricted to designated areas on base (e.g., smoke pits) and is not permitted while on the job site. However, smokeless tobacco is more easily concealed, and thus anti-tobacco policies may be more difficult to enforce or may be more easily overlooked by superiors. Smokeless tobacco may also be more common due to the danger of using combustible tobacco and lighters in some workplace environments, such as among maintainers who are often around flammable fuels. In contrast, Airmen in medical careers have limited opportunities to use tobacco due to their difficulty in accessing areas where smoking is permitted. Air Force medical treatment facility campuses, which include any clinic or hospital that provides medical or dental care for Department of Defense-eligible beneficiaries, are tobacco-free [20]. These campuses extend beyond the clinic or hospital to the surrounding parking structures, parking lots, lawns and other outdoor areas, making it inconvenient or impossible to use tobacco for any Airmen in a medical career field given that they cannot reach a designated smoking area in the time they are provided for a break. Additionally, they are less likely to be in open-air environments and are more likely to be patient-facing, and thus supplementing with non-cigarette tobacco products (e.g., smokeless) while working may be difficult.

Another important consideration is that Airmen in indoor/desk careers noted that they were less likely to receive breaks if they did not smoke cigarettes. This is consistent with smoking behavior among civilians working in the restaurant and hospitality industries, who also report cigarettes use as a way to access breaks [21]. This functional aspect of cigarette smoking in the workplace is imperative to consider when developing interventions or policy changes that will impact individuals working in these types of career fields. Further research is needed to assess how the provision of adequate breaks from work impact tobacco use decisions.

Further, Airmen in indoor/desk careers reported stress as a facilitator for smoking, consistent with research suggesting that stress is a facilitator for tobacco use among civilians [13,22]. Airmen tend to work long hours with fewer breaks, which could lead to increased stress [15]. There may also be fluctuations in stress at various careers stages (e.g., deployment [14]), which may also contribute to increased tobacco use [13]. Further, previous literature demonstrated that stress is a predictor of relapse [23] and has been linked to a reduced likelihood of resisting the urge to smoke [22]. Therefore, it seems particularly important to take job-related stress into consideration when tailoring smoking cessation interventions to Airmen, especially those working in indoor/desk careers.

The public health implications of tobacco use in the military are considerable since approximately 170,000 new recruits enter the military annually in one of the service branches [24]. Furthermore, the Department of Defense is the nation’s largest employer with 1.4 million active duty personnel [25], and costs associated with tobacco (e.g., medical care, hospitalizations, lost work days) was estimated at $600 million per year as of 2007 [26]. Given that approximately 200,000 individuals leave the military each year [27], with many continuing their tobacco dependence into their civilian lives, preventing and treating tobacco use among military personnel is of critical public health importance. Therefore, it will be essential to determine effective strategies to address these cultural aspects of tobacco use in different military careers.

Overall, it is clear that there are many career fields in the military where existing policies restricting tobacco use may not be enforceable or effective, and that tobacco has been established as part of the culture [28]. Given the vast differences between career fields in the military, tailoring tobacco prevention and cessation messages or resources to different career fields may be more effective than using a one-size-fits-all approach in order to change the narrative related to tobacco use. Tailoring prevention and cessation messages or resources to different career fields may also initiate a shift in the cultural acceptability of tobacco use among military personnel.

While it is important to examine how stricter tobacco control policies may reduce use among outdoor/hands-on career fields (similar to the effects of the tobacco free campuses seen around the military treatment facilities), it may also be critical to allocate resources to change the perceptions of descriptive and injunctive norms, and the acceptability of tobacco use. To implement this, the military could engage in social-norms campaigns which use various strategies (e.g., advertisements) to change public perceptions. Social-norms campaigns, such as those employed by the Truth Initiative, have shown promise among adolescents and young adults in college settings [14,29]. These types of intervention may be necessary to support tobacco free policies in the workplace.

Additionally, research assessing the impact of providing adequate breaks among those in positions where breaks are not typical is needed to better understand how this might impact tobacco use decisions. Lastly, it will be important to identify effective strategies to disseminate cessation resources to facilitate successful cessation among military members who use tobacco products.

### Limitations

One limitation of the present study is that we only collected data from the U.S. Air Force, and thus our findings may not be generalizable to other military branches or to other countries’ armed forces. However, the career fields in the Air Force are similar across branches, and thus one could reasonably generalize to the other branches of the military. Further, our study sample included only enlisted Airmen and findings may not be generalizable to officers, as literature suggested that tobacco use is different (i.e., much lower) among officers [4,30]. Finally, as focus groups were conducted anonymously to facilitate sharing of honest perspectives, detailed demographic and tobacco use history data were not collected. However, future research could examine potential demographic and tobacco use history differences to more fully understand the evolution of tobacco use in military career fields.

## 5. Conclusions

Despite these limitations, the current study is the first, to our knowledge, to identify differences related to the culture of tobacco product use among diverse career fields within the U.S. military. Quantitative evaluations of tobacco use by career field should be conducted to accumulate more evidence about prevalence of use, facilitators, and deterrents of tobacco use that will inform tobacco control programs and policies in the military. Future studies can expand upon this research to develop tailored interventions and adequate policies that may help to reduce tobacco use burden among military personnel.

## 6. Disclaimer

The opinions expressed on this document are solely those of the authors and do not represent an endorsement by or the views of the United States Air Force, the Department of Defense, the United States Government or the National Institutes of Health.

## Figures and Tables

**Table 1:** Identified Career Field Categories.

Career Field Category	Description	Example Career Fields
Outdoor/Hands-on Jobs	Career fields in which Airmen work in hands-on careers that often provide them direct and frequent access to outdoor spaces.	Careers on the flight line Careers in maintenance Civil engineering
Indoor/Desk Jobs	Career fields that require Airmen to work indoors, often at specific workstations.	Intel Air Traffic Control
Medical	Career fields in which Airmen work in medical facilities. While most Airmen in this career field category have direct patient care, some do not as they maintain the medical and facility equipment.	Clinicians Medical Technicians Technicians who maintain medical and healthcare setting system equipment
Security Forces	Career fields in which Airmen are often outside on base patrol or guard duty.	Police Special Forces

**Table 2:** Airmen’s Broad Perceptions of Tobacco Use in the Air Force and across Career Fields.

Perception	Exemplar Quote
Tobacco use is part of Air Force and military culture	“… because practically every career field…there are people smoking in it. It has been so engrained in military culture that they used to give cigarettes in MREs and not more than 25 years ago. So, to raise the Airmen up like, “Nobody in the military smokes. It is bad. You shouldn’t do it” and things like that, I feel like we are doing them a disservice because as soon as they get to their first duty station, things are much different.” (MTL, Keesler)
Tobacco use is not the same across career fields	“I think it depends on where you are and what job you do. So, if you’re doing it as a social thing in Tech School, maybe in medical, when you get to your first job, you’re gonna be embarrassed and to the point where you’re gonna want to quit or you are gonna hide and do it in private. But if you’re Security Forces, CE [Civil Engineering], anything like that, yeah, you’re gonna do it.” (MTL, Fort Sam) “I think the job itself also plays a big role ‘cause my last base, I worked on an aircraft, working on [the] flight line all the time, so we have 12-h shifts. People were working night shift. We were allowed to smoke in front of airplanes, out on the flight line, like each spot for each plane has its own smoke spot, like 200 yards away. So, everybody’s out there smoking. You just see smoke just rolling by because it either helps them stay awake long hours. So, there was a lot of people doing that. But yeah, these individuals here, they work in an office inside, so limited access.” (MTL, Goodfellow)
Tobacco use rates may be higher in career fields that work outdoors and/or engage in hands-on tasks than those than are more “desk” or indoor oriented	“It just depends on your career field. If you are in a career field where you are outside, like Maintenance and Security Forces or whatever, you are going to have more opportunities to smoke. If you are in an office setting or throughout the day than everybody else, then you are not going to be able to do that.” (MTL, Keesler) “Working inside, like you’re not gonna be able to smoke, but you’re already outside and I feel like you can just walk over there and just waste some time, mess around.” (MTL, Keesler)

**Table 3:** Exemplar Quotes Describing Facilitators and Deterrents of Tobacco Use by Career Field.

Career Category	Exemplar Quotes
Outdoor/Hands-On Jobs	“I’m prior maintenance, so if you don’t smoke, then there’s something wrong with you.” (MTL, Lackland, *accepted/normative nature of tobacco use*) “What we do in the hangar [use smokeless tobacco] directly correlates to what we’re going to be doing on the flight line in our jobs and we’re not going to have time for that [taking a break to smoke cigarettes].” (Technical Trainee, Sheppard, *accepted/normative nature of tobacco use & smokeless tobacco can be used while doing job*) “That’s why we [maintainers] dip, so we don’t have to stop.” (MTL, Goodfellow, *smokeless tobacco can be used while doing job*) “They say tobacco causes... dipping, tongue cancer; smoking, lung cancer. Well, 99 percent of the stuff that we do these days causes cancer. The jobs we do definitely cause cancer.” (Technical Trainee, Sheppard, *low perceptions of harm due to other occupational carcinogen exposures*)
Indoor/Desk Jobs	“A lot of the intels are introverted, so they don’t like talking to people. … And I feel like using tobacco might be one way that they cope with things ‘cause they don’t want to talk to you, they don’t want to come see us to deal with their problems. … So, I think that they just gravitated towards the negative coping methods.” (MTL, Goodfellow, *means to handle stress*) “I know for me if I didn’t smoke I would not take breaks, lunch, or anything, fact.” (MTL, Lackland, *means to get a break*)
Medical	“Bio—we’re medical, too, but we’re not in the same building. A lot of the people I work with smoke a lot. Just constantly go outside and smoke and every 20 min, they’re smoking.” (MTL, Fort Sam, *roles with outdoor access have opportunities to use*) “And I don’t feel like that’s the culture at all in medical. I was ashamed about it, almost to the point, like I don’t tell people that I do even now. Because the people who I was working with in medical made it to a point where it was like you were disgusting.” (MTL, Fort Sam, *not part of the culture*) “We’re just in a health field, so we all know that it’s not healthy to smoke, and then having our patient when we ask them, “Do you smoke? Do you drink?” One thing that always stands by me is like if I’m talking to someone and they smell like cigarettes, the perception of them is like, “I don’t really care what you’re telling me right now because you’re a smoker.” (TTI, Fort Sam, *use is seen as unprofessional & hard to hide use*)
Security Forces	“I think security forces uses smoking as a way to get breaks because we don’t get breaks. We don’t sit an office where you can take a 10-min break, or you can go up to the gym, or you can take lunch, or whatever. BAS means “bring a sandwich” in security forces, so if you don’t have—let’s say you’re on a patrol. You might have a little bit more leeway. But if you’re on a static post, on a flight-line post, you don’t get any time, so people I think take up smoking.” (MTL, Keesler, *smokeless tobacco can be used while doing job*) “In security forces, it tends to be dip. I see a lot more people do dipping than smoke because you have to be able to, like, you have to be physically fit, like master sergeant said, you have to be able to run and dip. I can’t sit here and smoke to you, but I can sit here and dip and talk to you” (TTI, Lackland, *smokeless viewed as not impacting function*) “I just know with the security forces a lot of people do it just to socialize. It’s the cool thing to do. Sometimes people do it, like I said I did it because I was bored on deployments. It gave me something to do.” (MTL, Lackland, *means for socialization*)

## Data Availability

The data presented in this study are available on request from the corresponding author, Melissa Little.
